# Editorial: Health Care Financing and Affordability in the Emerging Global Markets

**DOI:** 10.3389/fpubh.2016.00002

**Published:** 2016-01-21

**Authors:** Mihajlo Jakovljevic, Wim Groot, Kyriakos Souliotis

**Affiliations:** ^1^The Faculty of Medical Sciences, University of Kragujevac, Kragujevac, Serbia; ^2^Department of Health Services Research, CAPHRI, Faculty of Health, Medicine and Life Sciences, Maastricht University Medical Centre, Maastricht University, Maastricht, Netherlands; ^3^Top Institute Evidence-Based Education Research (TIER), Maastricht University, Maastricht, Netherlands; ^4^Faculty of Social and Political Sciences, University of Peloponnese, Tripoli, Greece

**Keywords:** health economics, emerging markets, third world, financing, government, affordability, low–middle income countries, BRICS

**The Editorial on the Research Topic**

**Health Care Financing and Affordability in the Emerging Global Markets**

During recent decades, global economic growth has been mostly driven by developing world economies. The ones with the most intensive pace of development were marked by Goldman Sachs as top-tier “emerging” markets led by BRICS (1) (Brazil, Russia, India, China, and South Africa) and N-11 (2) (Bangladesh, Egypt, Indonesia, Iran, South Korea, Mexico, Nigeria, Pakistan, the Philippines, Turkey, and Vietnam) countries. Compared to the past few centuries, the substantial novelty of world economic history is the bold surge in global South–South Cooperation and trade (3). Such changes inevitably reflect the global health arena (4). A number of issues previously limited to the established high-income economies became hot topics on the agendas of public health policy makers across these regions (Laaser). Major challenges continue to be population aging (5), rising incidence of prosperity diseases, lack of universal insurance coverage (6), and provision of just and equitable access to medical care among the poor, both in urban and rural communities (7). A large part of the difficulties faced by these societies can be attributed to inefficient resource allocation strategies in health care and unsatisfactory funding strategies (8).

The Frontiers research topic entitled “Health Care Financing and Affordability in the Emerging Global Markets” was created in order to tackle these core challenges across emerging global markets (Jakovljevic et al.). Outside of the BRICS markets, there were successful submissions referring to other dynamically developing Eastern European countries (Poland, Hungary, Czech Republic, and Ukraine), Balkan countries (Serbia, Albania, Republic of Srpska, Poland, and Bulgaria), Papua New Guinea representing Southeast Asia as well as ones in established OECD market economies, such as the USA, Japan, and Greece. Besides the two original research articles, the majority of published opinion style articles deal with crucial health economics and health policy challenges within the topic scope. Health policy considerations primarily focused on financing mechanisms and affordability of health care with a strong emphasis on cost-sharing mechanisms (Tambor et al.) and out-of-pocket payments (Atanasova et al.).

One of the two papers on BRICS reflected on the huge burden of non-communicable diseases in these countries and effectively associated with joint burden of communicable, infectious diseases in younger age groups (Jakovljevic and Milovanovic). Another one referred to the surprisingly sudden enlargement of BRICS’ share of global health spending (Jakovljevic). With regard to individual nations, health insurance coverage was challenged in a paper depicting contemporary momentum in rural India (Barik and Thorat).

A variety of articles dealt with issues on the health-care systems of the Balkans. Serbia, Greece, Republic of Srpska, Albania, and Bulgaria were serving as regional examples of the effects of transitional health reforms to neonatal care (Velickovic et al.), affordability of medicines (Petrusic and Jakovljevic), pharmaceutical marketing (Dickov), population aging (Stojkovic and Milovanovic), dentistry care (Kanjevac), burden of major prosperity illnesses such as COPD (Cupurdija), risk-sharing agreements for innovative therapies (Iskrov and Stefanov), effects of global recession to the pharmaceutical expenditure reduction (Souliotis et al.), social protection of vulnerable population groups (Arsenijevic et al.), and successes of WHO introduction of national health accounts system (Gajic-Stevanovic).

There were several articles describing developments on patient cost sharing and out-of-pocket payments in Central and Eastern European region (Tomini et al.). Although starting from different observation angles, authors ultimately arrive to the similar conclusion that growing income inequality and informal payments (Stepurko et al.) pose long-term challenges to the effective provision of medical services.

Evolution of Hungarian national efforts to eradicate informal payments was brought to us by Baji et al. Health expenditure landscape evolved alongside transitional reforms accordingly (Rancic et al). Distinguished contribution by Yamada et al. was dealing with effects of income and education on health-care disparity based on historical experience outsourcing from the US federal health system (Yamada et al.). Pioneering work in Papua New Guinea as the only Asian health system depicted in this topic was provided courtesy of Tsukahara et al. It gives a valuable insight into internal difficulties of this Southeast Asian country striving to achieve universal health coverage. The case of community health workers attempting to provide relief in childhood febrile episodes was used as an example of local difficulties (Tsukahara et al.).

In conclusion, editors would like to point out satisfactory response by broad professional audience worldwide. Topic impact and readership appears to be truly global, encompassing 15,150+ readers affiliated to the academia, industry, and regulatory authorities in all major world regions. Many of the articles have already attracted citations even before the topic closure. One of the major weaknesses in our attempt to foster strong professional discussion were relatively few contributions by the experts based in leading African and Latin American nations. Regardless of its undisputed success, our topic remains heavily dominated by Eastern European authors dealing with issues relevant primarily to this world region. Although we believe that we succeeded to spark a debate, further similar attempts in health economics of the emerging markets should focus on other key global regions. The Asian continent and People’s Republic of China, in particular, should be the main target of health financing and affordability research in years to come.

## Author Contributions

This editorial is committed to the final closure of research topic entitled Health Care Financing and Affordability in the Emerging Global Markets. MJ, WG, and KS have jointly and equally contributed to drafting manuscript and describing contributions out of which this research topic consisted. All authors deserve fully their authorship based on the intellectual content they provided and joint workflow during the topic development.

## Conflict of Interest Statement

The authors declare that the research was conducted in the absence of any commercial or financial relationships that could be construed as a potential conflict of interest.

## References

[1] O’Neill J Building Better Global Economic BRICS. Global Economics Paper No: 66. Goldman Sachs (2001). p. S1–15. Available from: http://www.goldmansachs.com/our-thinking/archive/archive-pdfs/build-better-brics.pdf

[2] Lawson S Heacock D Stupnytska A A look at the ‘Next 11’. In: O’Neill J, editor. Beyond the BRICs. The Goldman Sachs Group (2007). p. 159–64. Available from: http://www.goldmansachs.com/our-thinking/archive/archive-pdfs/brics-book/brics-chap-13.pdf

[3] Ross RJS Chan A From North-South to South-South: the true face of global competition. Foreign Aff (2002) 81:8–13. 10.2307/20033265

[4] Jakovljevic MB The key role of the leading emerging BRIC markets in the future of global health care. SJECR (2014) 15(3):139–43. 10.5937/sjecr15-6706

[5] Wilson D Burgi C Carlson S Population Growth and Ageing in the BRICs. Goldman Sachs Global Economics, Commodities and Strategy Research, BRICs Monthly (2011). p. 1–4. Available from: http://www.goldmansachs.com/our-thinking/archive/archive-pdfs/population-growth-ageing-brics.pdf

[6] Marten R McIntyre D Travassos C Shishkin S Longde W Reddy S An assessment of progress towards universal health coverage in Brazil, Russia, India, China, and South Africa (BRICS). Lancet (2014) 384(9960):2164–71. 10.1016/S0140-6736(14)60075-1 24793339 PMC7134989

[7] Marmot M Friel S Bell R Houweling TA Taylor S Commission on social determinants of health. Closing the gap in a generation: health equity through action on the social determinants of health. Lancet (2008) 372(9650):1661–9. 10.1016/S0140-6736(08)61690-6 18994664

[8] Jakovljevic MB. Resource allocation strategies in Southeastern European health policy. Eur J Health Econ (2013) 14(2):153–9. 10.1007/s10198-012-0439-y 23143312

